# Lysosomal dysfunction increases exosome-mediated alpha-synuclein release and transmission

**DOI:** 10.1016/j.nbd.2011.01.029

**Published:** 2011-06

**Authors:** Lydia Alvarez-Erviti, Yiqi Seow, Anthony H. Schapira, Chris Gardiner, Ian L. Sargent, Matthew J.A. Wood, J. Mark Cooper

**Affiliations:** a University Department of Clinical Neurosciences, Institute of Neurology, University College London, UK; b Department of Physiology, Anatomy and Genetics, University of Oxford, UK; c Nuffield Department of Obstetrics and Gynaecology, University of Oxford, John Radcliffe Hospital, UK

**Keywords:** Alpha-synuclein, Exosome, Transmission, Lysosomal inhibition, Ammonium chloride, Bafilomycin A1

## Abstract

Alpha-synuclein aggregation plays a central role in Parkinson's disease pathology. Direct transmission of alpha-synuclein from pathologically affected to healthy unaffected neurons may be important in the anatomical spread of the disease through the nervous system. We have demonstrated that exosomes released from alpha-synuclein over-expressing SH-SY5Y cells contained alpha-synuclein and these exosomes were capable of efficiently transferring alpha-synuclein protein to normal SH-SY5Y cells. Moreover, the incubation of cells with ammonium chloride or bafilomycin A1 to produce the lysosomal dysfunction recently reported in Parkinson's disease led to an increase in the release of alpha-synuclein in exosomes and a concomitant increase in alpha-synuclein transmission to recipient cells. This study clearly demonstrates the importance of exosomes in both the release of alpha synuclein and its transmission between cells and suggests that factors associated with PD pathology accelerate this process. These mechanisms may play an important role in PD pathology and provide a suitable target for therapeutic intervention.

## Research Highlights

► Exosomes released from cells contain α-synuclein. ► Exosomes transfer α-synuclein protein between cells. ► Lysosomal defects seen in Parkinson's disease increase exosomal α-synuclein release. ► Lysosomal dysfunction increases α-synuclein transfer mediate by exosomes.

## Introduction

Parkinson's disease (PD) is a progressive neurodegenerative disorder characterised pathologically by the loss of dopaminergic neurons in the substantia nigra pars compacta and the presence of Lewy bodies (LB) which are intra-cytoplasmic inclusions containing high levels of alpha-synuclein (Spillantini et al., 1997). The discovery of mutations (A53T, A30P, E46K) and multiplications of the alpha-synuclein gene in familial forms of PD further demonstrate the importance of alpha-synuclein to PD pathology (Lee and Trojanowski 2006). Host-to-graft propagation of alpha-synuclein-positive Lewy-like pathology has recently been demonstrated in long-term mesencephalic transplants in PD patients (Li et al., 2008; Kordower et al. 2008). *In vitro* experiments co-culturing over-expressing cells with non-expressing and neuronal precursor cells also showed cell-to-cell transmission of alpha-synuclein (Desplats et al., 2009). These studies support the notion that alpha-synuclein can be directly transmitted from pathologically affected to healthy unaffected neurons leading to progression of the disease process through the nervous system. This could be an explanation of the step-wise progression of the disease pathology and the involvement of anatomically distinct pathways. Recently, a study described the secretion of alpha-synuclein in association with membrane vesicles of composition and biophysical properties consistent with their identification as exosomes (Emmanoulidou et al., 2010).

Exosomes are membrane-bound vesicles of endocytic origin released by numerous cell types and found in abundance in body fluids (Simpson et al., 2008) where they act as natural carriers of mRNA, miRNA and proteins (Schorey and Bhatnagar 2008). Exosomes have been associated with prion protein release from cultured non-neuronal and neuronal cells (Fevrier et al., 2004; Vella et al., 2007); moreover, exosomes released from prion-infected neuronal cells were efficient initiators of prion propagation in uninfected recipient cells.

We examined exosomes released from SH-SY5Y cells stably over-expressing WT alpha-synuclein to determine if they contained alpha-synuclein protein and whether exosomes can mediate alpha-synuclein transfer between neuronal cells. The inter-cellular transfer of alpha-synuclein may not in itself be sufficient to propagate PD pathology and other factors may play a role. Given the observation that lysosomal function is essential for alpha-synuclein metabolism and the evidence of lysosomal dysfunction in PD brains (Alvarez-Erviti et al., 2010) we assessed whether lysosomal dysfunction could influence alpha-synuclein release and transmission.

## Material and methods

All reagents were obtained from Sigma Aldrich (Dorset, UK) or Merck (Nottingham, UK) unless otherwise stated.

### Cell cultures

Normal SH-SY5Y cell and a clone constitutively expressing full length human wild type alpha-synuclein with a C-terminal HA tag (alpha-synuclein-HA) were grown under standard conditions with the addition of G418 (0.4 mg/ml) for maintenance of the clone (Chau et al., 2009). Lysosomal inhibition was achieved by incubating cells with 20 mM ammonium chloride for up to 7 days, or with 200 nM bafilomycin A1 for up to 72 h. SH-SY5Y cells were differentiated by treatment with 10 μM all-trans retinoic acid for 7 days.

### Cell proliferation

Equal cell numbers of treated and untreated cells were seeded after various treatments and cell proliferation rates were analysed by the Celltiter Blue kit (Promega).

### Exosome purification and cell treatment

Foetal calf serum used for exosome production was centrifuged at 25,000*g* for 90 minutes at 4 °C before the preparation of medium. Cells used for exosome isolation were 80–90% confluent, culture medium was changed 24 h before the isolation of exosomes. Twenty four hour conditioned medium from 10 × 10 cm plates of cells (70–80% confluent) was collected and centrifuged for 10 minutes at 1000*g* followed by 12,000*g* to exclude cell debris, and exosomes pelleted from the post-12,000*g* supernatant by centrifugation at 120,000*g* for 1 h (Quah and O'Neill 2005).

Exosome pellets were resuspended in 100 μl growth medium and incubated with normal SH-SY5Y cells (70% confluent 35 mm plate) for 16 h.

### Exosome immunoprecipitation

Fifty microliters of Protein-A Sepharose beads (Sigma P9424) were diluted in 500 μl PBS containing BSA (2 mg/ml) and incubated overnight at 4 °C. The beads were washed 3 times with PBS and resuspended in 100 μl anti-flotillin-1 antibody (1/100 dilution in PBS/BSA 2 mg/ml, rabbit polyclonal, Abcam) or anti-tubulin (1/500 dilution in PBS/BSA 2 mg/ml, rabbit polyclonal, Abcam) and incubated at 4 °C for 4 h. The beads were washed 3 times with PBS, and purified exosomes were added to the beads in 200 μl of PBS and incubated for 3 h at 4 °C. After incubation, the beads were washed 3 times with PBS and eluted in 0.1% SDS for Western blot analyses.

### Alpha-synuclein ELISA analysis

To quantify total alpha-synuclein release by cells 24 h conditioned medium from 2 × 10 cm plates was concentrated with Amicon Ultra-15 Centrifugal Filter Unit (Millipore). The medium was sonicated for 20 minutes (sonicator bath), and total released alpha-synuclein was assessed using an alpha-synuclein ELISA kit (USCN Life) as per manufacturer's protocol. Results were normalised to the number of cells on the original plates.

### Extraction of alpha-synuclein

Cell pellets were sequentially extracted in high salt (HS, 50 mmol/l Tris, 750 mmol/l NaCl, 5 mmol/l EDTA), HS/Triton (1% Triton) and SDS/urea (8 mol/l of urea, 2% SDS) and insoluble material pellet by centrifugation at 60,000*g* as previously described (Waxman and Giasson 2008).

### Electron microscopy and Nanoparticle Tracking Analysis (NTA)

For electron microscopy exosome preparations were incubated with 4% osmium tetraoxide for 30 minutes at 4 °C, then applied onto a copper grid and stained with 1% phosphotungstic acid.

NTA was carried out using the Nanosight LM10-HS system (NanoSight, Amesbury, UK) on exosomes resuspended in PBS at a concentration of approximately 3 μg of protein/ml and were further diluted between one and five hundred-fold for analysis. The system focuses a laser beam through a suspension of the particles of interest. These are visualised by light scattering, using a conventional optical microscope aligned normally to the beam axis which collects light scattered from every particle in the field of view. A 20–60 second video records all events for further analysis by NTA (Nanoparticle Tracking Analysis) software. The Brownian motion of each particle is tracked between frames, ultimately allowing calculation of the size via application of the Stokes–Einstein equation.

### Western blots, immunocytochemistry and antibodies

The NuPAGE gel system (Invitrogen) was used for all protein separations using 4–12% polyacrylamide gel. Cells were lysed in 10 mM Tris pH 7.4 buffer containing 0.1% SDS, a protease inhibitor cocktail and DNase (Promega, Southampton UK) and incubated for 1 h at 37 °C. SDS–PAGE separations, Western blot and ECL detection protocols have been described previously (Chau et al., 2009).

Primary antibodies used for Westerns were mouse anti-Lamp-1 antibody (Abcam 1/5000), mouse anti-Alix (Abcam 1/500), rabbit anti-flotillin-1 (Abcam 1/1000), mouse anti-HA (Covance 1/5000), mouse anti-alpha-synuclein (Zymed 1/1000), rabbit anti-actin (Abcam 1/5000). Secondary antibodies used were HRP-conjugated anti-mouse or anti-rabbit (DAKO, Ely, UK).

For immunofluorescence SH-SY5Y cells were seeded on coverslips and immunostained using the anti-alpha-synuclein antibody (1:200), anti-HA antibody (1:1000) as previously described (Chau et al., 2009). Images were taken using an Axiophot fluorescence microscope and KS400 software (Zeiss, Welwyn Garden City, UK) with standardised exposure times. Confocal analysis was performed using a Zeiss confocal microscope (LSM 510 Meta), the sections were first examined using low-magnification lenses (× 10 and × 20) and photographs were taken at higher magnifications (× 63 oil immersion objective).

### Statistics

Statistical analyses of the data were performed using SPSS program 16.0 by using the non-parametric Kruskal–Wallis test followed by the Mann–Whitney *U* test. The data are presented as means ± standard deviations and represents results from at least 3 independent experiments.

## Results

### Exosomes released by SH-SY5Y cells contained and transferred alpha-synuclein

**Fig. 1:**
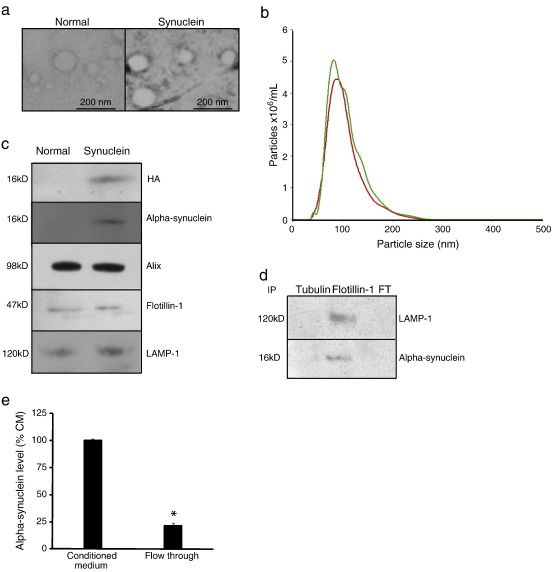
Analysis of exosomes released by SH-SY5Y. Characterisation of exosomes isolated from conditioned medium (CM) from normal and alpha-synuclein over-expressing SH-SY5Y cells: a, Electron micrograph of phosphotungstic acid-stained exosomes; scale bar = 200 nm. b, NTA analysis of particle diameters in exosome preparations from CM from normal (green line) or alpha synuclein expressing (red line) SH-SY5Y cells. c, Western blot analysis of exosome markers (LAMP-1, Alix and flotillin-1) and alpha-synuclein (alpha-synuclein and HA). d, Conditioned medium from alpha synuclein expressing cells was immunoprecipitated (IP) with anti-tubulin or anti-flotillin-1 antibodies. All the eluted IP protein and 40% (15 μg) of the flow through (FT) were loaded and probed with anti-LAMP-1 and anti-alpha-synuclein antibodies. e, ELISA quantitation of alpha-synuclein levels in the flow through after IP with flotillin-1 presented as a percentage of the level in the CM.

Exosomes were isolated from normal and WT alpha-synuclein over-expressing SH-SY5Y conditioned medium (CM) using an established ultracentrifugation protocol (Quah and O'Neill 2005). Exosomes obtained from the ultracentrifuge pellets were examined by electronic microscopy and by NTA. Both techniques demonstrated that a homogenous population of exosomal vesicles with a size distribution peaking at a diameter of 93 and 99 nm for normal and alpha-synuclein expressing cells respectively (Fig. 1a and b) could be readily isolated and identified. Western blot analysis confirmed the presence of the exosomal proteins, LAMP-1, flotillin-1 and Alix (Simpson et al., 2008) in exosome preparations from both sets of cells. However, alpha-synuclein protein was detectable only in exosomes from cells over-expressing alpha-synuclein-HA (Fig. 1c). This finding was consistent with the undetectable levels of endogenous alpha-synuclein protein in normal SH-SY5Y cells (data not shown). Antibodies against flotillin-1 were used to immunoprecipitate exosomes from CM from alpha-synuclein over-expressing cells. The immunoprecipitate was positive for LAMP-1 and alpha-synuclein and the flow through medium was negative for both proteins (Fig. 1d). Quantitation of alpha-synuclein levels in conditioned medium and flow through using ELISA analysis (Fig. 1e) confirmed the majority of the alpha-synuclein was associated with the exosomes.

**Fig. 2:**
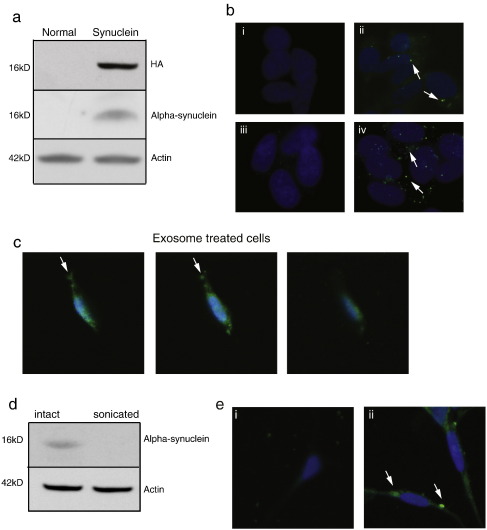
Analysis of the inter-cellular transfer of alpha synuclein by exosomes, a. Western blot analysis of alpha-synuclein levels (using anti-alpha-synuclein and HA antibodies) relative to actin in normal SH-SY5Y cells following overnight treatment with exosomes isolated from CM from normal and alpha-synuclein over-expressing SH-SY5Y cells. b, Immunofluoresence staining for alpha-synuclein (i and ii, anti-alpha-synuclein antibody; iii and iv, anti-HA antibody, arrows) in normal SH-SY5Y cells (i and iii) and after 16 h exposure to exosomes isolated from CM from alpha-synuclein over-expressing cells (ii and iv). c, Serial confocal Z-projection images (4 μm) of normal SH-SY5Y cells 16 h after incubation with exosomes isolated from WT alpha-synuclein over-expressing cells, anti-alpha-synuclein antibody (green) and DAPI (blue). d. Western blot analysis of alpha-synuclein levels (alpha-synuclein antibody) relative to actin in normal SH-SY5Y cells following overnight treatment with intact and sonicated exosomes isolated from CM from alpha-synuclein over-expressing SH-SY5Y cells. e. Immunofluoresence staining of alpha-synuclein (alpha-synuclein antibody, green) relative to DAPI (blue) in differentiated normal SH-SY5Y cells grown under control conditions (i) or 16 h after treatment with exosomes isolated from CM from alpha-synuclein over-expressing cells (ii).

To investigate whether alpha-synuclein could be transmitted from one cell to another via exosomes we isolated exosomes from CM from normal and alpha-synuclein over-expressing SH-SY5Y cells and incubated them with normal SH-SY5Y cells for 16 h. Only the SH-SY5Y cells incubated with exosomes isolated from alpha-synuclein expressing cells showed the presence of detectable alpha-synuclein on Western blot (Fig. 2a) and immunocytochemical analyses (Fig. 2b and c).This was detected with both anti-alpha-synuclein and anti-HA antibodies confirming the transmission from the donor cells (Fig. 2b) and confocal analysis confirmed its intracellular location (Fig. 2c). Disruption of exosomes by sonication (Stoeckl et al., 2006) prior to their incubation with recipient cells prevented the transfer of alpha-synuclein (Fig. 2d), indicating efficient transfer of alpha-synuclein to recipient cells required intact exosomes. This transfer was not unique to dividing cells as we were also able to confirm the exosomal dependent transfer of alpha-synuclein to differentiated SH-SY5Y cells (Fig. 2e).

### Influence of lysosomal inhibition upon alpha-synuclein levels

**Fig. 3:**
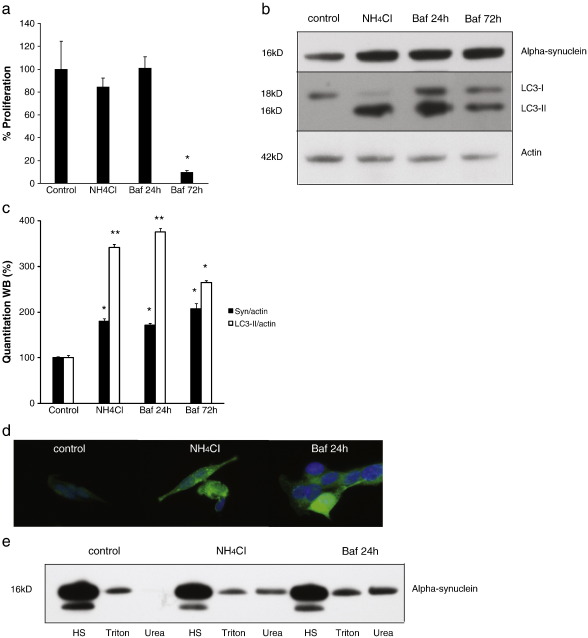
Influence of lysosomal inhibition on alpha-synuclein levels. Analysis of alpha-synuclein overexpressing cells under normal conditions (control) or after treatment with ammonium chloride or bafilomycin A1. a, Influence of ammonium chloride (20 mM) treatment for 7 days and bafilomycin A1 (200 nM) treatment for 24 and 72 h upon cell proliferation as determined by the Celltiter Blue kit in alpha synuclein over-expressing SH-SY5Y cells. Statistical analysis compared to controls *p < 0.05. b and c, Western Blot analysis of alpha-synuclein (alpha-synuclein antibody) and LC3-I and II levels relative to actin after 7 days ammonium chloride or 24 and 72 h bafilomycin A1 treatment. Statistical analysis compared to controls *p < 0.05, **p < 0.01. d, Immunofluoresence staining for alpha-synuclein (anti-HA antibody, green) after 7 days of treatment with ammonium chloride or 24 h of treatment with bafilomycin A1. e, Western Blot analysis of alpha-synuclein levels in SH-SY5Y cells treated with ammonium chloride or bafilomycin A1. Cells were sequential extracted with high salt (HS), HS/Triton (Triton) and SDS/urea (urea), and 3%, 3% and 20% of each extract respectively were separated and detected with an anti-alpha-synuclein antibody.

Alpha-synuclein protein is degraded predominantly through lysosomal pathways therefore requiring intact lysosomal function (Paxinou et al., 2001). In an attempt to mimic the abnormal lysosomal function described in PD (Alvarez-Erviti et al., 2010) we inhibited lysosomal function in normal and WT alpha-synuclein over-expressing SH-SY5Y cells with ammonium chloride for 7 days or with the vacuolar H + ATPase inhibitor bafilomycin A1 for 24 and 72 h (Martinez-Vicente et al., 2008). There were no adverse effects on cell survival with the exception of prolonged bafilomycin treatment which caused marked cell loss (Fig. 3a). The level of the autophagosomal marker LC3-II was increased with these treatments consistent with decreased degradation of autophagic vacuoles associated with lysosomal dysfunction (Fig. 3b and c). Relative to untreated cells alpha-synuclein levels (corrected to actin levels) increased significantly following ammonium chloride (increased 79.6% ± 6.3) and bafilomycin (increased 71.3% ± 3.8 after 24 h, 107.5% ± 11.2 after 72 h) treatment in over-expressing cells (Fig. 3b–d) but remained undetectable in normal cells (data not shown). The impact of these treatments upon aggregate formation was investigated using the differential solubility of alpha-synuclein. Cells were sequentially extracted in high salt, 1% triton and SDS/urea and the relative levels of alpha-synuclein assessed in each fraction. While the majority of alpha-synuclein was extracted in high salt medium in all conditions there was clearly evidence of a triton insoluble pool of alpha-synuclein following both ammonium chloride and bafilomycin treatments that was not seen in the controls (Fig. 3e). This suggested that lysosomal dysfunction led to evidence of insoluble alpha-synuclein consistent with the presence of aggregates; however, while the immunocytochemical images were consistent with alpha-synuclein accumulation there was no clear evidence of aggregates.

### Lysosomal dysfunction increased alpha-synuclein release and transmission

**Fig. 4:**
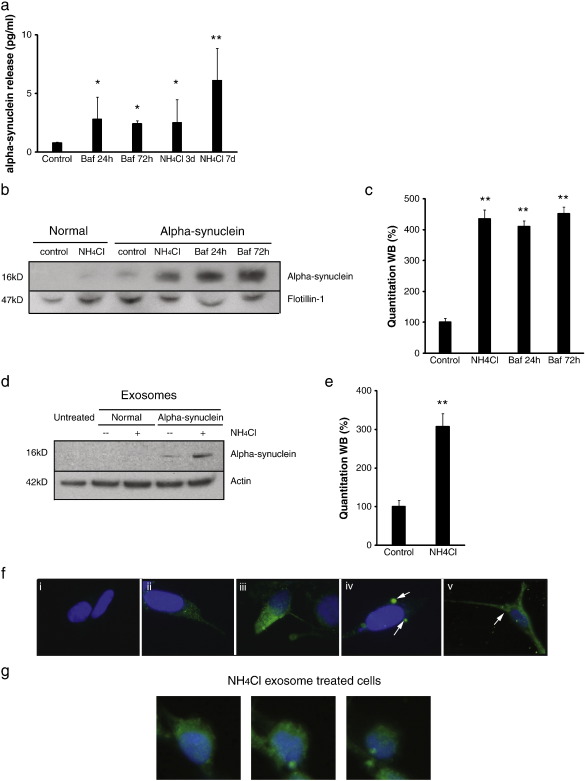
Influence of lysosomal inhibition upon exosome-mediated alpha-synuclein transmission to normal cells. a, ELISA quantitation of total alpha-synuclein release under control conditions or after ammonium chloride or bafilomycin A1 treatment. Data compared to controls, *p < 0.05, **p < 0.01. b and c, Western Blot analysis of exosomes isolated from CM from normal and alpha-synuclein over expressing cells grown under control conditions or after ammonium chloride (20 mM for 7 days) or bafilomycin A1 (200 nM) treatment. Alpha-synuclein levels (alpha-synuclein antibody) were evaluated relative to an exosome marker, Flotillin-1. Statistical analysis compared to controls **p < 0.01. d and e, Western Blot analysis of alpha-synuclein levels (alpha-synuclein antibody) relative to actin in normal SH-SY5Y cells following 16 h incubation with exosomes isolated from CM from normal and alpha-synuclein over-expressing SH-SY5Y cells grown in the absence or presence of ammonium chloride (7 days). Statistical analysis compared to controls **p < 0.01. f, Immunofluoresence staining of alpha-synuclein (alpha-synuclein antibody) in (i) normal untreated SH-SY5Y cells, and (ii) normal SH-SY5Y cells after 16 h incubation with exosomes isolated from alpha-synuclein over-expressing cells, (iii–v) alpha-synuclein over-expressing cells after ammonium chloride treatment, (iv and v) demonstrating peri-nuclear alpha-synuclein accumulation (arrows). All studies were with dividing SH-SY5Y cells except for panel v where the recipient cells had been differentiated for 7 days. g, Serial confocal Z-projections of normal SH-SY5Y cells 16 h after incubation with exosomes isolated from WT alpha-synuclein over-expressing cells treated with ammonium chloride, anti-alpha-synuclein antibody (green) and DAPI (blue).

Lysosomal dysfunction caused a dramatic increase in total alpha-synuclein release into the cell medium (increased 300–620% at all time points assessed, Fig. 4a). The isolation of exosomes from the conditioned medium demonstrated this marked increase in alpha-synuclein levels was also apparent in the exosomes after exposure to ammonium chloride for 7 days (435% ± 29.8) or bafilomycin A1 for 24 h (397.5% ± 18.2) or 72 h (451.9% ± 21.6) (Fig. 4b and c).

Following incubation of the exosomes released from alpha-synuclein over-expressing cells inhibited by ammonium chloride with normal SH-SY5Y cells, the level of alpha-synuclein present in the recipient cells was markedly increased compared with that seen in experiments performed without lysosomal inhibition (increased 307.1 ± 34.1 %, Fig. 4d and e). This was also evident upon immunofluorescent analysis with an increase in both the intensity of alpha-synuclein staining (Fig. 4f) and number of recipient cells containing detectable levels of alpha-synuclein (96% and 38% of recipients cells after incubation with exosomes from ammonium chloride treated and untreated cells, respectively). Confocal microscopy analysis confirmed the presence of alpha-synuclein throughout the cell consistent with an intracellular location (Fig. 4g). Moreover, the presence of alpha-synuclein positive inclusions (seen in 0.2% of cells after incubation with exosomes from untreated cells) was also more apparent in cells incubated with exosomes from ammonium chloride treated cells (seen in 3.6% of cells) (Fig. 4f, panels iii and iv). These observations were not only restricted to the transfer of alpha-synuclein to mitotic cells as similar results were obtained when exosomes were incubated with differentiated SH-SY5Y cells (Fig. 4f, panel v).

## Discussion

While there is clear evidence that alpha-synuclein plays an important role in PD pathology, its exact role in pathogenesis remains unclear. Alpha-synuclein is prone to aggregation leading to its presence in LB; however, the debate as to whether soluble protofibrils or aggregates of alpha-synuclein are the pathological entity remains to be resolved. The Braak staging of PD pathology suggests that there is a progression of LB pathology from the brainstem to the cortex consistent with pathological propagation along specific neural pathways (Braak et al., 2003). The recent finding of Lewy body inclusions in embryonic dopaminergic nerve cells implanted into the striatum of patients with PD (Li et al., 2008; Kordower et al., 2008) has given fresh impetus to the importance of alpha-synuclein propagation in PD pathology.

While alpha-synuclein is predominantly a cytosolic protein associated with dopamine metabolism, extracellular alpha-synuclein has been observed in CSF and plasma in humans (Borghi et al., 2000; El-Agnaf et al., 2003). The release of alpha-synuclein from cells via exocytosis has been previously reported in SH-SY5Y cells over-expressing human alpha-synuclein (Lee et al., 2005) and associated with membrane vesicles whose identity is compatible with exosomes (Emmanoulidou et al., 2010), and we have confirmed this observation. The possibility of pathological alpha-synuclein propagation between cells was further strengthened when alpha-synuclein was recently shown to be transferred to neighbouring cells, via a process involving endocytosis (Desplats et al., 2009). However to date the precise mechanisms of alpha-synuclein release and uptake by recipient cells remains ill defined.

Exosomes have been previously associated with pathological prion protein transmission from neuronal and non-neuronal infected cell lines to uninfected recipient cells (Fevrier et al., 2004; Alais et al., 2008). We have demonstrated that alpha-synuclein released from SH-SY5Y cells can be recovered by a centrifugation protocol used to purify exosomes (Quah and O'Neill 2005). While exosomes do not have any unique characteristic feature, a combination of features is accepted as evidence of their identity (Simpson et al., 2008). The characterisation of vesicle preparations in this study revealed an electron microscopic appearance, vesicle size profile and the presence of LAMP-1, flotillin-1 and Alix proteins, all of which are indicative of an exosomal identity. These results are in agreement with observations from cells with inducible expression of alpha-synuclein (Emmanoulidou et al., 2010). We were also able to immunoprecipitate the exosomes from alpha-synuclein over-expressing cells which pulled down the majority of the alpha-synuclein in the medium consistent with the role of exosomes in the extracellular release of alpha-synuclein. The isolated exosomes were capable of transferring alpha-synuclein to naive cells; however, sonicated exosomes were no longer able to efficiently do this outlining the importance of the exosome in this transfer mechanism. This transfer was not restricted to dividing cells as similar data were obtained with differentiated SH-SY5Y recipient cells demonstrating the exosome mediated transfer of alpha-synuclein could be effective in differentiated neurones. Confocal microscopy analysis was consistent with the intracellular localisation of alpha-synuclein within the recipient cells. While not all cells stained positive for the transferred alpha-synuclein, occasional cells showed the presence of large inclusions. These could represent vesicle bound alpha-synuclein or the presence of aggregates but were not abundant enough for definitive analysis.

While it is apparent that alpha-synuclein is transferred between cells, its relevance to normal physiology or pathology is not clear. Equally, while the transmission of alpha-synuclein could lead to the propagation of this protein throughout the brain, its transfer between cells may not be sufficient in itself to transmit the pathology associated with PD. It is therefore important to demonstrate that this process can be involved in the propagation of aggregated alpha-synuclein in a manner similar to that suggested for prion diseases (Fevrier et al., 2004; Alais et al., 2008). It is possible that the transfer of alpha-synuclein from one cell to the next initiates a cell stress response ultimately leading to alpha-synuclein aggregation and further transmission to neighbouring cells (Desplats et al., 2009). Equally the biochemical abnormalities found in PD brains could exacerbate the propagation of a pathological form of alpha-synuclein which, when transferred to neighbouring cells, acts to ‘seed’ aggregate formation.

In an attempt to model the generation of an aggregate prone form of alpha-synuclein we investigated the effect of lysosomal inhibition. Lysosomal function has been reported to be decreased in PD patients (Chu et al., 2009; Alvarez-Erviti et al., 2010) and alpha-synuclein is known to require the lysosome for its degradation (Paxinou et al., 2001). In an attempt to mimic a defect of intracellular protein handling in PD we inhibited lysosomal function in the exosome donor cells using ammonium chloride or bafilomycin A1. Lysosomal inhibition by ammonium chloride for 3 and 7 days and bafilomycin for 24 h and 72 h all dramatically increased alpha-synuclein release from the SH-SY5Y cells. However, this was only associated with significant cell death in cells treated with bafilomycin for 72 h consistent with exosome mediated alpha-synuclein release and not a consequence of cell death. While lysosomal inhibition increased the cellular content of alpha-synuclein, we were unable to detect any clear evidence of intra-cellular protein aggregates; however, there was an increase in triton insoluble alpha-synuclein suggesting the presence of aggregates. The increase in cellular alpha-synuclein levels was associated with a dramatic increase in exosomal alpha-synuclein release which led to greater transmission of alpha-synuclein to recipient cells and a greater number of cells containing alpha-synuclein inclusions. These data suggest that the increased exosomal release of alpha-synuclein may be influenced by the increase in intracellular alpha-synuclein consistent with the suggestion that in neurons exosomes may play a direct role in the removal of unwanted proteins (Putz et al., 2008).

We hypothesise that changes to lysosomal function in PD can accelerate exosomal alpha-synuclein release and propagation to neighbouring cells and a concomitant increase in alpha-synuclein inclusion formation. We believe that this cellular model is a useful approach to test whether other biochemical changes observed in PD can lead to the generation of pathological forms of alpha-synuclein in donor cells that can be propagated via exosomes to other cells and play a role in PD pathogenesis.

## References

[bb0005] Alvarez-Erviti L., Rodriguez-Oroz M.C., Cooper J.M., Ferrer I., Obeso J.A., Schapira A.H. (2010). Chaperone-mediated autophagy markers in Parkinson's disease brains. Arch. Neurol..

[bb0010] Alais S., Simoes S., Baas D., Lehmann S., Raposo G., Darlix J.L., Leblanc P. (2008). Mouse neuroblastoma cells release prion infectivity associated with exosomal vesicles. Biol. Cell.

[bb0015] Borghi R., Marchese R., Negro A., Marinelli L., Forloni G., Zaccheo D., Abbruzzese G., Tabaton M. (2000). Full length alpha-synuclein is present in cerebrospinal fluid from Parkinson's disease and normal subjects. Neurosci. Lett..

[bb0020] Braak H., Del Tredici K., Rüb U., de Vos R.A., Jansen Steur E.N., Braak E. (2003). Staging of brain pathology related to sporadic Parkinson's disease. Neurobiol. Aging.

[bb0025] Chau K.Y., Ching H.L., Schapira A.H., Cooper J.M. (2009). Relationship between alpha synuclein phosphorylation, proteasomal inhibition and cell death: relevance to Parkinson's disease pathogenesis. J. Neurochem..

[bb0030] Chu Y., Dodiya H., Aebischer P., Olanow C.W., Kordower J.H. (2009). Alterations in lysosomal and proteasomal markers in Parkinson's disease: relationship to alpha-synuclein inclusions. Neurobiol. Dis..

[bb0040] Desplats P., Lee H.J., Bae E.J., Patrick C., Rockenstein E., Crews L., Spencer B., Masliah E., Lee S.J. (2009). Inclusion formation and neuronal cell death through neuron-to-neuron transmission of alpha-synuclein. Proc. Natl Acad. Sci. USA.

[bb0045] El-Agnaf O.M., Salem S.A., Paleologou K.E., Cooper L.J., Fullwood N.J., Gibson M.J., Curran M.D., Court J.A., Mann D.M., Ikeda S., Cookson M.R., Hardy J., Allsop D. (2003). Alpha-synuclein implicated in Parkinson's disease is present in extracellular biological fluids, including human plasma. FASEB J..

[bb0050] Emmanouilidou E., Melachroinou K., Roumeliotis T., Garbis S.D., Ntzouni M., Margaritis L.H., Stefanis L., Vekrellis K. (2010). Cell-produced alpha-synuclein is secreted in a calcium-dependent manner by exosomes and impacts neuronal survival. J. Neurosci..

[bb0055] Fevrier B., Vilette D., Archer F., Loew D., Faigle W., Vidal M., Laude H., Raposo G. (2004). Cells release prions in association with exosomes. Proc. Natl Acad. Sci. USA.

[bb0060] Kordower J.H., Chu Y., Hauser R.A., Freeman T.B., Olanow C.W. (2008). Lewy body-like pathology in long-term embryonic nigral transplants in Parkinson's disease. Nat. Med..

[bb0065] Lee H.J., Patel S., Lee S.J. (2005). Intravesicular localization and exocytosis of alpha-synuclein and its aggregates. J. Neurosci..

[bb0070] Lee V.M., Trojanowski J.Q. (2006). Mechanisms of Parkinson's disease linked to pathological alpha-synuclein: new targets for drug discovery. Neuron.

[bb0075] Li J.Y., Englund E., Holton J.L., Soulet D., Hagell P., Lees A.J., Lashley T., Quinn N.P., Rehncrona S., Björklund A., Widner H., Revesz T., Lindvall O., Brundin P. (2008). Lewy bodies in grafted neurons in subjects with Parkinson's disease suggest host-to-graft disease propagation. Nat. Med..

[bb0080] Martinez-Vicente M., Talloczy Z., Kaushik S., Massey A.C., Mazzulli J., Mosharov E.V., Hodara R., Fredenburg R., Wu D.C., Follenzi A., Dauer W., Przedborski S., Ischiropoulos H., Lansbury P.T., Sulzer D., Cuervo A.M. (2008 Feb). Dopamine-modified alpha-synuclein blocks chaperone-mediated autophagy. J. Clin. Invest..

[bb0085] Paxinou E., Chen Q., Weisse M., Giasson B.I., Norris E.H., Rueter S.M., Trojanowski J.Q., Lee V.M., Ischiropoulos H. (2001). Induction of alpha-synuclein aggregation by intracellular nitrative insult. J. Neurosci..

[bb0090] Putz U., Howitt J., Lackovic J., Foot N., Kumar S., Silke J., Tan S.S. (2008). Nedd4 family-interacting protein 1 (Ndfip1) is required for the exosomal secretion of Nedd4 family proteins. J. Biol. Chem..

[bb0095] Quah B.J., O'Neill H.C. (2005). The immunogenicity of dendritic cell-derived exosomes. Blood Cells Mol. Dis..

[bb0100] Schorey J.S., Bhatnagar S. (2008). Exosome function: from tumor immunology to pathogen biology. Traffic.

[bb0105] Simpson R.J., Jensen S.S., Lim J.W. (2008). Proteomic profiling of exosomes: current perspectives. Proteomics.

[bb0110] Spillantini M.G., Schmidt M.L., Lee V.M., Trojanowski J.Q., Jakes R., Goedert M. (1997). Alpha-synuclein in Lewy bodies. Nature.

[bb0115] Stoeckl L., Funk A., Kopitzki A., Brandenburg B., Oess S., Will H., Sirma H., Hildt E. (2006). Identification of a structural motif crucial for infectivity of hepatitis B viruses. Proc. Natl Acad. Sci. USA.

[bb0120] Vella L.J., Sharples R.A., Lawson V.A., Masters C.L., Cappai R., Hill A.F. (2007). Packaging of prions into exosomes is associated with a novel pathway of PrP processing. J. Pathol..

[bb0125] Waxman E.A., Giasson B.I. (2008). Specificity and regulation of casein kinase-mediated phosphorylation of alpha-synuclein. J. Neuropathol. Exp. Neurol..

